# Exploiting the Pleiotropic Antioxidant Effects of Established Drugs in Cardiovascular Disease

**DOI:** 10.3390/ijms160818185

**Published:** 2015-08-05

**Authors:** Sebastian Steven, Thomas Münzel, Andreas Daiber

**Affiliations:** 1 2nd Medical Clinic, University Medical Center of the Johannes Gutenberg-University, Mainz 55131, Germany; E-Mails: Sebastiansteven@gmx.de (S.S.); tmuenzel@uni-mainz.de (T.M.); 2 Center for Thrombosis and Hemostasis, University Medical Center of the Johannes Gutenberg-University, Mainz 55131, Germany

**Keywords:** cardiovascular disease, endothelial dysfunction, oxidative stress, inflammation, dipeptidyl peptidase-4 inhibitors, glucagon-like peptide analogs

## Abstract

Cardiovascular disease is a leading cause of death and reduced quality of life worldwide. Arterial vessels are a primary target for endothelial dysfunction and atherosclerosis, which is accompanied or even driven by increased oxidative stress. Recent research in this field identified different sources of reactive oxygen and nitrogen species contributing to the pathogenesis of endothelial dysfunction. According to lessons from the past, improvement of endothelial function and prevention of cardiovascular disease by systemic, unspecific, oral antioxidant therapy are obviously too simplistic an approach. Source- and cell organelle-specific antioxidants as well as activators of intrinsic antioxidant defense systems might be more promising. Since basic research demonstrated the contribution of different inflammatory cells to vascular oxidative stress and clinical trials identified chronic inflammatory disorders as risk factors for cardiovascular events, atherosclerosis and cardiovascular disease are closely associated with inflammation. Therefore, modulation of the inflammatory response is a new and promising approach in the therapy of cardiovascular disease. Classical anti-inflammatory therapeutic compounds, but also established drugs with pleiotropic immunomodulatory abilities, demonstrated protective effects in various models of cardiovascular disease. However, results from ongoing clinical trials are needed to further evaluate the value of immunomodulation for the treatment of cardiovascular disease.

## 1. Oxidative Stress in Cardiovascular Disease and Inflammation

### 1.1. Introduction

The majority of cardiovascular diseases are accompanied by an imbalance between the formation of reactive oxygen species (ROS, including superoxide, hydrogen peroxide as well as precursor products peroxynitrite or hypochlorous acid) and antioxidant enzymes [1,2], leading to a deviation from the steady state [3]. More recent evidence suggests that adverse redox signaling and oxidative stress are not only side effects of the progression of cardiovascular disease but even potent triggers of their development and pathogenesis [4,5]. According to the “kindling radical” hypothesis (or “bonfire” concept), the formation of ROS may trigger the activation of additional sources of ROS in certain disease conditions or during the aging process [6,7]. According to recent reports, vascular dysfunction in general, but hypertension and coronary artery disease may also be linked to inflammation or low-grade activation of the immune system [8,9]. Uncoupling of endothelial nitric oxide synthase (eNOS) is a hallmark of most cardiovascular disease [10,11] and endothelial dysfunction in coronary and peripheral vessels, measured by acetylcholine-dependent plethysmography or flow-mediated dilation, is an early predictor of cardiovascular events [12,13]. eNOS function is regulated by many different factors such as subcellular localization, calcium levels, binding of different co-factors (e.g., BH_4_, FAD, FMN, NADPH, zinc), and other proteins (e.g., calmodulin, heat shock proteins). Regulation of eNOS activity by so-called “redox switches” is of great interest for the present review—the oxidative depletion of tetrahydrobiopterin (BH_4_), oxidative disruption of the dimeric eNOS complex by oxidation of the zinc-sulfur complex, *S*-glutathionylation of a cysteine in the reductase domain, and adverse phosphorylation at Thr495/Tyr657, as well as ROS-triggered increases in levels of the endogenous eNOS inhibitor asymmetric dimethylarginine (ADMA) (for detailed review see [6,7]). Another focus of research in the cardiovascular field is the “repair” of vascular damage by improvement of the function of endothelial progenitor cells by drugs with antioxidant and other pleiotropic properties [14] or infusion of these cells after a severe insult such as myocardial infarction [15,16]. This topic will only be touched on with some examples but not explored in detail. The major part of this review will discuss antioxidant therapeutic interventions that prevent eNOS uncoupling, thereby normalizing endothelial function in particular and improving cardiovascular disease in general. We will emphasize the importance of low-grade inflammation in the development of endothelial dysfunction and cardiovascular disease and discuss the contribution of specific inflammatory cells and their cytokine profiles to the development and progression of cardiovascular disease.

### 1.2. Inflammation, Oxidative Stress, and Endothelial Dysfunction

**Figure 1:**
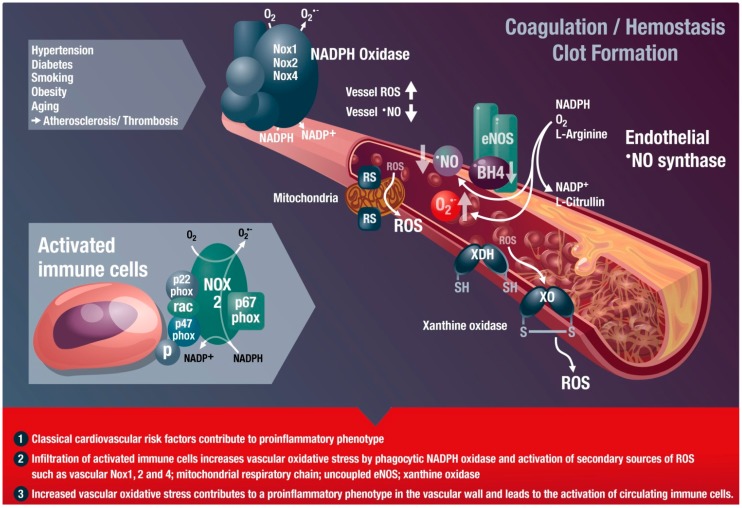
Inflammatory cells, vascular dysfunction, and atherothrombosis. The scheme illustrates the activation of immune cells and recruitment to vascular tissues by classical cardiovascular risk factors, leading to activation of secondary vascular ROS sources such as NADPH oxidase (Nox1, Nox2, and Nox4), xanthine oxidase (conversion of the dehydrogenase (XDH) to the oxidase (XO) form), mitochondria (via mitochondrial redox switches (RS)), and uncoupled eNOS (oxidative depletion of tetrahydrobiopterin (BH4) and other redox switches), all of which contribute to vascular dysfunction. Immune cells such as monocytes need the functional phagocyte-type NADPH oxidase (Nox2) in order to infiltrate vascular tissues. ROS produced by this activated Nox2 from infiltrated immune cells will activate secondary vascular ROS sources in a redox-sensitive fashion (for review see [19,30]). These processes lead to late-stage cardiovascular complications such as atherosclerosis with plaque formation and thrombosis. Modified from [8]. With permission by Bentham Science Publisher. Copyright © 2014, Eureka Science Ltd.

The first description of the role of oxidative stress in the development and progression of cardiovascular disease in an experimental model of hypercholesterolemia was published by Harrison and Ohara [17,18]. According to the abovementioned concept of “kindling radicals” or the “bonfire” hypothesis, the initial formation of superoxide (e.g., from phagocytic NADPH oxidases of infiltrated leukocytes) and subsequent formation of peroxynitrite most likely triggers further damage such as eNOS uncoupling, converting this beneficial nitric oxide synthase into a detrimental superoxide-producing enzyme (Figure 1) [6,8]. Likewise, ROS from infiltrated immune cells can activate or induce expression of vascular cell oxidases such as Nox1, Nox2, or Nox4 (isoform specific catalytic subunits of NADPH oxidases), or mediate the oxidative conversion of the xanthine dehydrogenase to the oxidase form [6,19]. This ROS-induced ROS formation is well known for cross-activation of mitochondrial ROS formation by dysfunctional mitochondria [20]. Mitochondrial ROS formation and release can be stimulated by thiol-oxidation in different mitochondrial structures (e.g., mitochondrial permeability transition pore constituents such as cyclophilin D, p66^shc^ or monoamine oxidases); xanthine dehydrogenase is converted to the oxidase form by oxidation of critical thiol residues; the protective action of eNOS to produce ^•^NO is switched to adverse superoxide formation by oxidative depletion of BH4, adverse phosphorylation by redox-activated kinases, *S*-glutathionylation ,or oxidative disruption of the zinc-sulfur complex at the dimer binding interface, called the “uncoupling” process. These changes (increased vascular oxidative stress and release of inflammatory signaling molecules) will lead to endothelial cell activation and priming for the adhesion of additional immune cells as well as platelets and switch the vasodilatory, antiaggregatory, and antiatherosclerotic phenotype of the endothelium to a vasoconstrictory, proaggregatory, and proatherosclerotic one.

As described above, ROS formation is not only a side effect of cardiovascular diseases but directly contributes to the disease progression in many ways. For example, the induction of endothelial dysfunction by oxidative modification of eNOS or its cofactors as well as redox stimulation of inflammatory cascades fosters the progression of these cardiovascular diseases (Figure 1). Most immune cells express high levels of functional NADPH oxidases and are capable of producing ROS at much higher levels than vascular cells [21,22]. The important role of phagocytic NADPH oxidase in this process was demonstrated by the fact that white blood cells with dysfunctional Nox2 were not able to infiltrate the vascular wall and induce vascular oxidative stress and damage [22,23], e.g., by oxidative conversion of xanthine dehydrogenase to the oxidase form, uncoupling of eNOS, or stimulation of mitochondrial ROS formation and release by specific redox switches [6]. On the other hand, there is clear evidence that ROS formation *per se* contributes to a pro-inflammatory phenotype, since mitochondrial superoxide/hydrogen peroxide formation has the ability to activate immune cells [24,25,26]. ROS play an important role in inflammation and tissue damage [27]. There is also increasing evidence of a close interaction between vascular oxidative stress and inflammation during the aging process, leading to a vicious cycle in the aging vasculature [28]. By this crosstalk, infiltrated immune cells promote vascular oxidative stress, lead to endothelial cell activation, and prime the endothelium for the adhesion of additional leukocytes and platelets [8], which is of great importance for aging-associated endothelial dysfunction [29]. *Vice versa*, oxidative stress is a hallmark of all cardiovascular disease and will also lead to endothelial cell activation, priming for adhesion and infiltration of immune cells as well as activation of these infiltrated immune cells. Accordingly, most cardiovascular disease displays a low-grade inflammatory phenotype of the vasculature.

Different immune cells have been reported to contribute to the development of cardiovascular disease, but since they interact with each other, the individual impact of each cell type on the development of cardiovascular disease remains elusive. The contribution of B- and T-cells to the development of hypertension by angiotensin-II infusion was demonstrated by RAG-1^−/−^ mice [22]. Likewise, the onset of angiotensin-II induced hypertension and vascular oxidative stress was also attenuated by removal of myelomonocytic cells [23]. Of note, immune suppressive treatment of patients with rheumatoid arthritis or psoriasis was associated with a reduction of systolic blood pressure [31], underlining the clinical impact of a direct contribution of the immune system to vascular dysfunction.

### 1.3. Chronic Autoimmune Diseases Associated with Cardiovascular Disease

Chronic autoimmune diseases such as systemic lupus erythematodes, rheumatoid arthritis, and severe psoriasis are associated with an increased risk for cardiovascular events [32,33,34,35]. Importantly, psoriasis was defined as an independent risk factor in addition to the classical cardiovascular risk factors such as smoking, obesity, and diabetes [36]. The European League against Rheumatism even recommends the management of cardiovascular risk in inflammatory arthritis in their guidelines [37]. As an early predictor of cardiovascular events, impaired vascular function was observed under chronic inflammation, which was evident in patients with rheumatoid arthritis by a significant increase in the intima-media thickness [38] or impaired endothelial function measured by flow-mediated dilatation in patients with psoriasis [39]. Therefore, recent clinical trials have demonstrated that increased cardiovascular mortality in patients with chronic inflammatory disease can be managed by targeting specific cytokines or activation of specific immune cells, e.g., in psoriasis the IL-17/IL-23 axis [40,41,42], in systemic lupus erythematodes IL-17A signaling [43], and in rheumatoid arthritis the IL-6, TNF-α, and IL-17A cascades [44,45]. These data provide a feasible link between cardiovascular disease and the chronic autoimmune diseases (also reviewed in [32,34]).

## 2. Classical Antioxidants and New Strategies to Modulate Oxidative Stress

### 2.1. Classical Antioxidants

Based on the oxidative stress concept in cardiovascular, neurodegenerative, metabolic, and inflammatory disease [5,7,8], numerous studies were conducted. *In vitro* and animal studies of these diseases were performed in order to characterize the cytoprotective or therapeutic benefit of antioxidants and to promote phytochemicals, functional foods, and antioxidant (vitamin) supplements. However, antioxidants have failed to show any therapeutic benefit in most large clinical trials that were conducted according to modern standards [46], such as HOPE (Heart Outcome Prevention Evaluation) and HOPE-TOO (Heart Outcome Prevention Evaluation—The Ongoing Outcomes), which demonstrated that vitamin E causes more heart failure and left heart decompensation [47,48,49,50] (for review see [51]). A prospective study with vitamin C in post-menopausal women with diabetes mellitus even demonstrated an increased incidence of cardiovascular events and mortality under antioxidant therapy [52]. The SAINT I trial investigated the therapeutic benefit of the synthetic antioxidant, NXY-059, in acute ischemic stroke but failed to show any neuroprotective effect [53]. According to Bjelakovic and coworkers, meta-analysis of 68 randomized trials with 232,606 participants revealed that the use of lipid-soluble antioxidants without medical indication may even increase mortality in adults [54]. Another meta-analysis of 14 randomized trials with 170,525 individuals by the same author demonstrated a similar trend—lipid-soluble antioxidants increased the mortality of gastrointestinal cancer patients [55]. However, other meta-analyses support the beneficial effects of vitamin C in specific disease conditions or disease-associated impairment of functional parameters, e.g., on the survival of women with breast cancer [56] or on endothelial function in patients with atherosclerosis, diabetes, and heart failure [57].

These large-scale clinical trials on chronic oral antioxidant supplementation are contrasted by multiple small cohort studies with acute (parenteral) administration of antioxidants with highly beneficial effects on the surrogate parameters of disease (e.g., endothelial dysfunction) in chronic smokers or patients with diabetes or coronary artery disease [12,58,59,60] (for review see [51]). The advantage of parenteral administration of water-soluble antioxidants is that high plasma concentrations of the antioxidant are achieved [61], thereby omitting the complications of oral absorption (time-lag, limited capacity) and insufficient compliance. High-dose intravenous infusion of vitamin C also improved endothelial function in patients with Kawasaki disease [62], kidney dysfunction [63], hypertension [64], liver cirrhosis, and portal hypertension [65]. Moreover, parenteral application of vitamin C has also proven to have clinical effects in patients with allergies [66], sudden hearing loss [67], breast cancer, infection, and pancreatitis [68].

A positive example of the beneficial effect of chronic antioxidant therapy is vitamin D. Lack of vitamin D is endemic in the human population and epidemiological data indicate that deficiency of this vitamin is associated with cardiovascular disease [69]. There is some evidence from interventional trials demonstrating that supplementation of vitamin D is beneficial to endothelial function [70,71], blood pressure [72], and cardiac hypertrophy [73,74] in humans. Furthermore, a recent Cochrane analysis revealed that vitamin D supplementation significantly reduces cardiovascular mortality in elderly people [75]. Nevertheless, further large-scale randomized placebo-controlled clinical trial are needed to elucidate the cardiovascular protective effects of vitamin D. In contrast to other vitamins, deficiency of vitamin D is very common, especially in older individuals [76], which might be the explanation for the beneficial effects of vitamin D, especially on cardiovascular disease in the elderly [77].

Based on the disappointing results of most large-scale clinical trials (HOPE, HOPE-TOO) [47,49] (reviewed in [51]), with chronic oral antioxidants supplementation the question arises whether the oxidative stress hypothesis in pathogenesis and disease progression is wrong? We think the answer is no and possible explanations for the lack of clinical efficacy of antioxidants in these studies might be that: (1) vitamins C and E act as pro-oxidants; (2) the coronary artery disease of the patients included in the studies is already irreversible; (3) the coronary artery disease patients are already being treated with drugs displaying antioxidant properties (for example, ACE inhibitors and angiotensin type 1 receptor blockers; see also Section 3); (4) chronic antioxidant therapy inhibits intrinsic ischemic preconditioning, which relies on ROS formation; and (5) oral vitamin treatment does not result In high enough concentrations of the antioxidants at the place of oxidative stress (summarized from [5]). Another important drawback of classical antioxidants may be the slow reaction between O_2_^•−^ and vitamins C and E (with rate constants of 3.3 × 10^5^ and 4.9 × 10^3^ M^−1^·s^−1^, respectively, compared with 1.9 × 10^10^ M^−1^·s^−1^ for the reaction between ^•^NO and O_2_^•−^) [78]. Finally, in most of the abovementioned large-scale clinical trials on the use of chronic oral antioxidant supplementation, the compliance of the patients was not controlled (e.g., by measurement of plasma levels of the antioxidants). The important role of controlled antioxidant plasma levels became evident in the EPIC Norfalk study, demonstrating that vitamin C concentrations in the blood inversely correlate with all-cause mortality in healthy volunteers [79]. In addition, there was an inverse correlation between circulating vitamin C concentrations and risk of stroke as reported by a meta-analysis [80]. According to a concept put forward by Lykkesfeldt and colleagues [81], better results of antioxidant therapy might be expected under conditions of antioxidant deficiency (e.g., for vitamin C and E) [82], or often encountered for vitamin D [76]. Some of these reasons would favor a proper prediagnosis of patients for blood levels of antioxidants, strict monitoring of this parameter during antioxidant therapy, and acute infusion of vitamin C in accordance with the observations and advantages of parenteral use discussed above. According to a recent review on the use of antioxidants in translational medicine, future antioxidant strategies will not be based on the classical antioxidant vitamins (apart from some acute situations with subclinical deficiencies as well as parenteral instead of oral therapy) but rather on the activation of endogenous antioxidant enzyme systems, inhibition of critical ROS sources (e.g., NADPH oxidase), the repair of oxidatively damaged structures, or site-directed antioxidant approaches [51].

### 2.2. New Antioxidant Strategies

Direct and cell organelle-specific targeting of ROS formation is a new promising strategy. A prominent example is scavenging mitochondrial ROS by mitochondria-targeted antioxidants such as mitoquinone (mitoQ) [83,84], a quinone that is coupled to a triphenylphosphonium group to facilitate mitoQ accumulation in mitochondria by up to 10,000-fold. MitoQ showed beneficial effects in an animal model and in human cells from patients with chronic obstructive pulmonary disease [85], improved nitrate-tolerance-associated side effects of nitroglycerin therapy in rats [86], and normalized endothelial function and cardiac hypertrophy in stroke-prone spontaneously hypertensive rats [87]. Moreover, MitoQ showed neuroprotective effects in experimental amyotrophic lateral sclerosis [88], suppressed NLRP3 inflammasome-mediated inflammatory cytokines in a murine colitis model [89], beneficially influenced nephropathy in diabetic mice [90], and prevented cardiac ischemia-reperfusion injury in rats [84]. According to recent data, another mitochondria-targeted antioxidant, mitoTEMPO, prevented adverse effects of angiotensin-II in experimental hypertension [91]. Similar beneficial effects have been reported for the use of mitochondria-targeted SOD mimetics such as Mn(III) 5,10,15,20-tetrakis(*N*-methylpyridinium-2-yl)porphyrin (MnTM-2-PyP^5+^) in various disease models [92,93]. Several compounds of the class of mitochondria-targeted antioxidants are currently in late phase clinical trials (for review see [92,93,94,95]; for ongoing clinical trials visit www.clinicaltrials.gov). A limitation of the use of mitochondria-targeted antioxidants might be that viable mitochondria with intact membrane potential are required for their mitochondrial accumulation, which could interfere with their uptake, especially in dysfunctional, ROS-producing mitochondria. A similar idea provides the basis for endothelium-targeted antioxidants. Shuvaev *et al.* demonstrated that targeting of SOD, but not catalase, to the endothelium reversed angiotensin II-induced endothelial dysfunction [96], nicely demonstrating that O_2_^•−^ is a more harmful species in the vascular system than H_2_O_2_. The antioxidant enzymes SOD and catalase were conjugated with an antibody against PECAM-1 (platelet/endothelial cell adhesion molecule-1) to ensure endothelial binding. Another strategy would be to covalently bind SOD mimetics or antioxidants to heparin [97], which will lead to the binding of these antioxidant compounds to heparin-binding sites on the endothelial cell layer [98,99].

Modulation/regulation of endogenous antioxidant defense systems or ROS sources by miRNAs (e.g., antagomirs), epigenetic drugs (e.g., modulators of histone acetyl transferases or deacetylases), or phytochemicals are other completely new antioxidant strategies [100,101,102,103,104,105,106]. Resveratrol, a phytochemical antioxidant, was previously regarded as a direct ROS scavenger, but more recent data revealed that it mostly acts via indirect antioxidant mechanisms [107], e.g., by modulation of gene expression via miRNAs, epigenetic modifications, and direct effects on proteins of the DNA repair machinery [108,109,110]. Besides resveratrol, there are hundreds of these phytochemical antioxidant compounds, in many cases with proven therapeutic effects and in some cases even with mechanistic explanations for these observed clinical effects. One prominent example is *Ginkgo biloba*, which has been in clinical use for a long time, especially for dementia therapy but also for its positive effects on cardiovascular disease [111].

Other antioxidant strategies are only briefly mentioned here (we refer to the respective review articles, e.g., [51]) and are based on: (1) the inhibition of disease-relevant ROS sources such as inhibitors of NADPH oxidase (Nox) enzymes [112,113,114,115] or xanthine oxidase [116,117]; (2) upregulation of the endogenous antioxidant defense system such as Nrf2 agonists [118]; and (3) repair of oxidatively damaged protein structures as exemplified by activators of heme-deficient or oxidized soluble guanylyl cyclase [119,120,121]. According to Stocker and colleagues, an antioxidant should ideally be recycled by cellular reducing systems, act catalytically to prevent its consumption, or induce endogenous antioxidant defense systems rather than act as a direct scavenger (for review see [122]).

## 3. Antioxidants 2.0—Pleiotropic Antioxidant Effects of Established Drugs

It would be beyond the scope of this review to discuss all of the important representatives of this group of drugs, so the following will be limited to some examples of new function for old drugs. The pleiotropic antioxidant effects of these compounds are characterized by different modes of action (as already described above): (1) induction of intrinsic antioxidant systems; (2) inhibition of Nox2-dependent ROS formation; and (3) direct ROS scavenging activity. It remains to be established whether some of these pleiotropic antioxidant effects are just a consequence of the primary pharmacological action of the drugs (e.g., lowering of blood pressure). At least in some of the examples the primary pharmacological action of the drugs can be excluded since data were obtained either in cell culture or even with isolated enzymatic systems.

### 3.1. Statins, ACE-Inhibitors, and AT1-Receptor Blockers

Angiotensin-converting enzyme inhibitors, type 1 angiotensin II receptor antagonists, statins, and many other cardiovascular drugs display pleiotropic indirect antioxidant properties (e.g., inhibition of Nox enzymes and secondary to this prevention of eNOS uncoupling) [123,124]. In more detail, angiotensin-converting enzyme inhibitors and type 1 angiotensin II receptor antagonists increase the bioavailability of ^•^NO by decreased breakdown of bradykinin and activation of the corresponding B2 receptor [125]. They also prevent the activation of the phagocytic and vascular Nox2 enzyme and thereby decrease cellular superoxide, hydrogen peroxide, and peroxynitrite levels [126]. Inhibition of angiotensin-II signaling decreases oxidative stress since angiotensin-II via its receptor leads to the formation of diacylglycerol, a potent endogenous trigger of NADPH oxidase activity [127]. In addition, these drugs confer potent anti-inflammatory effects by interfering with the adhesion of monocytes to the endothelium [128] and even improving the severity of adjuvant arthritis [129]. In summary, angiotensin-converting enzyme inhibitors and type 1 angiotensin II receptor antagonists promote a vasodilatory, antithrombotic, and antiproliferative milieu and improve the function of endothelial progenitor cells [130]. These protective mechanisms might also explain their benefit for the therapy of patients with heart failure [131]. Statins obviously target exactly the same pathophysiological parameters. In patients with cardiovascular disease, statins reduce vascular inflammation and atherothrombosis, which causes cardiovascular events like myocardial infarction [132,133,134]. Statins reduce NADPH oxidase activity in a Rac1-dependent mechanism [135,136] and improve the bioavailability of ^•^NO through increased levels of the eNOS cofactor BH4, decreased levels of the endogenous eNOS inhibitor asymmetric dimethyl-l-arginine, decreased caveolin-1 activity, improved activating eNOS phosphorylation, and upregulation of eNOS mRNA [137]. The beneficial pleiotropic effects of statins are probably based on the induction of the Nrf2-heme oxygenase-1 system [138,139] but improvement of the function of endothelial progenitor cells could also contribute to their protective profile [140].

### 3.2. Nebivolol, Hydralazine, and Pentaerythrityl Tetranitrate (PETN)

One of the first known antihypertensive drugs was hydralazine, which is today mainly used for the treatment of pre-eclampsia [141]. However, it experienced a “revival” when the company NitroMed introduced their combination drug BiDil containing hydralazine and isosorbide dinitrate. This combination therapy showed an impressive decrease in mortality in African-Americans with severe heart failure, who responded poorly to ACE inhibitors and other standard medications (the study design was based on data from V-HeFT (Vasodilator Heart Failure Trial) and A-HeFT (African-American Heart Failure Trial) [142,143,144]. According to our previous observations, hydralazine is a highly efficient peroxynitrite scavenger and prevents tyrosine nitration [145,146], which may at least contribute to its beneficial effects on nitroglycerin-induced nitrate tolerance [147] and potentially isosorbide dinitrate-associated side effects. Based on these data, we postulate a direct antioxidant property of hydralazine by scavenging peroxynitrite, a potentially harmful oxidant. This provides the rationale for the beneficial effects of the hydralazine/isosorbide dinitrate combination to prevent side effects of the organic nitrate under chronic therapy (e.g., endothelial dysfunction [148]). Recent data support a reaction between peroxynitrite and dihydralazine sulfate [149]. However, there is also evidence for the indirect antioxidant effects of hydralazine by induction of hypoxia-inducible factor-1α, vascular endothelial growth factor, and angiogenesis by inhibition of prolyl hydroxylases [150]. Among other antihypertensive drugs, hydralazine has been found to possess pleiotropic antioxidant effects in patients beyond the direct blood pressure lowering effects [151]. Also, protective effects of hydralazine on endothelial progenitor cell function were reported [152].

The third generation beta-blocker nebivolol was reported to induce vascular nitric oxide formation via stimulation of eNOS activity in *ex vivo* studies [153,154,155], providing the rationale for improved ^•^NO bioavailability in patients with essential hypertension [156]. In this clinical study a combination therapy of nebivolol/bendrofluazide, in contrast to atenolol/bendrofluazide treatment, improved ^•^NO bioavailability despite a similar degree of blood pressure lowering. We could detect eNOS-stimulating effects of nebivolol neither in cultured endothelial cells nor in hypertensive mice when comparing wild-type controls with eNOS knockout mice (unpublished data, Karbach *et al.* and Daiber). We have previously shown that nebivolol, in contrast to metoprolol and atenolol, prevents eNOS uncoupling and induction of phagocytic NADPH oxidase activity in white blood cells, vascular oxidative stress, and endothelial dysfunction in hyperlipidemic Watanabe (WHHL) rabbits [157]. In a subsequent study we characterized nebivolol as a potent Nox2 inhibitor in hypertensive rats as well as isolated cells, which are not shared by first- and second-generation beta-blockers [158]. Nebivolol directly interferes with the assembly of Nox2 and cytosolic subunits p47^phox^, p67^phox^, and rac1 in the cytoplasmic membrane, suggesting that Nox2 inhibition leads to reduced superoxide formation, prevents eNOS uncoupling and breakdown of nitric oxide by reaction with superoxide, and finally ameliorates endothelial function. This concept goes hand in hand with human data in which nebivolol normalized oxidative stress in hypertensive patients and led to reduced oxidative degradation of nitric oxide [159]. Finally, nebivolol improved the function of early endothelial progenitor cells in experimental myocardial infarction, which could also contribute to its beneficial clinical profile [160].

After the development of pentaerithrityl tetranitrate (PETN) for the U.S. market, it was abandoned, but then used for many years in the former Eastern German Republic. After the reunion of Germany, PETN became the best-selling nitrate on the German market. PETN is the only organic nitrate in clinical use devoid of induction of nitrate tolerance, endothelial dysfunction, and other nitrate-associated side effects in volunteers [161,162] and patients with coronary artery disease [163,164]. The molecular explanation for the beneficial effects of PETN, not shared by other nitrates, is the induction of heme oxygenase-1 [165,166,167,168] in a Nrf2-dependent fashion [169]. PETN also induced extracellular superoxide dismutase [170], prevented vascular complications in experimental diabetes and hypertension [165,169], prevented the progression of atherosclerosis in a rabbit model [171], and inhibited platelet aggregation in heart failure [172]. In contrast to other organic nitrates, PETN improved the function (migration and incorporation) of endothelial progenitor cells and decreased their NADPH oxidase activity *ex vivo* and *in vivo* in humans and rats [173,174,175]. In addition, PETN therapy leads to the regulation of more than 1200 genes and upregulates several cardio-protective transcription factors, whereas nitroglycerin, also a nitrovasodilator, regulated approximately 500 genes different from those regulated by PETN [176]. More recently, PETN was shown to induce heritable epigenetic changes envisaged by H3K27 acetylation, H3K4 trimethylation, and transcriptional activation of eNOS, MnSOD, glutathione peroxidase-1, and heme oxygenase-1, all of which lead to reduced blood pressure in female offspring of PETN-treated hypertensive rats [177]. Of note, these beneficial effects were neither shared by other organic nitrates nor by the ^•^NO donors tested in this study. The ongoing CAESAR trial (Clinical efficacy study of Pentalong for pulmonary hypertension in heart failure; EudraCT Number: 2009-015059-26) will show whether the potent antioxidant and vasculoprotective effects of PETN can be translated to patients with pulmonary hypertension as a result of heart failure.

### 3.3. Gliptins and Glucagon-Like Peptide-1 (GLP-1) Analogs Display Antioxidant and Anti-Inflammatory Properties

Dipeptidyl peptidase-4 (DPP-4) is an exopeptidase also known as CD26. N-terminal dipeptides are cleaved from alanine- and proline-rich proteins [178]. Besides DPP-4, the family of DPPs consists of several members: DPP-1–DPP-4, DPP-6–DPP-9, quiescent cell proline dipeptidase (QPP), and fibroblast activation protein (FAP) [178,179]. DPP-4 has a wide range of functions, the best characterized of which is the degradation of incretins (glucagon-like peptide-1 (GLP-1) and gastric inhibitory polypeptide GIP) [179]. Furthermore, DPP-4 cleaves non-incretin peptides, possesses non-enzymatic function, and interacts with membrane bound proteins as a chaperone [179]. In various tissues DPP-4 is expressed on the surface of endothelial cells, epithelial cells, and inflammatory cells (monocytes, lymphocytes, dendritic cells, and natural killer (NK) cells) [180,181,182,183].

GLP-1 is an incretin hormone released from L-cells in the intestine after food uptake [184,185]. In the context of glucose homeostasis, circulating GLP-1 binds to its receptor, which is expressed on pancreatic beta-cells, but also on cardiomyocytes, endothelial cells, and inflammatory cells. The GLP-1 receptor belongs to the family of G-protein-coupled receptors. After binding of GLP-1 to its receptor, cAMP levels rise and insulin release is stimulated. On pancreatic alpha-cells, GLP-1 reduces glucagon release (for review see [186]). In summary, GLP-1 is involved in glycemic control, which makes it an attractive target for treatment of diabetes [187,188]. Derived from the prolucagon gene, GLP-1 (7–36-amide) and GLP-1 (7–37) are secreted. Due to rapid degradation of GLP-1 to GLP-1 (9–36-amide) by DPP-4, the half-life of GLP-1 is below 2 min [189,190]. There are two pharmacological strategies for using the GLP-1 effects on glucose metabolism in diabetic patients: (1) inhibition of DPP-4 by gliptins to increase GLP-1 levels and (2) supplementation of modified GLP-1, which resists degradation by DPP-4. At the time of this review five DPP-4 inhibitors are approved by the European Medicines Agency (EMA) (vildagliptin, alogliptin, sitagliptin, linagliptin, and saxagliptin) for treatment of type 2 diabetes mellitus, also reflected by the Global Guideline for Type 2 Diabetes [191]. GLP-1 analogs are represented by liraglutide and exenatide, also reflected by the Global Guideline for Type 2 Diabetes [191].

Besides their potent effects on glycemic control in diabetic patients, research of the last years revealed their effects on several other cell types and tissues. *In vivo* and *in vitro* studies demonstrated the beneficial effects of DPP-4 inhibitors on cardiovascular disease [192,193], but also in diseases like psoriasis [194], hepatic steatosis [195], or stroke [196]. Interestingly, pathogenesis of all of these diseases has oxidative stress and most likely inflammation in common. Finally, DPP-4 inhibitors improved the number and “homing” of endothelial progenitor cells in animal [197,198] and human [199] studies, but the number of publications on this association is still low. GLP-1 analogs seem to share these effects on endothelial progenitor cells [200].

Oeseburg *et al.* published evidence for a protective effect of DPP-4 inhibition on oxidative stress-induced DNA damage and cellular senescence in Zucker diabetic fatty rats [201]. The authors propose elevated GLP-1 to be responsible since the effect could be blocked by exendin fragment 9–39, which is a GLP-1 receptor antagonist. According to this study, induction of the antioxidant enzymes heme oxygenase-1 and NADPH dehydrogenase (quinone) via protein kinase A activation are responsible for the beneficial effects [201]. The mentioned study is an excellent example of the difficulty of differentiating between the direct GLP-1 effects and the GLP-1-independent effects of DPP-4 inhibition. Others detected reduced oxidative stress under DPP-4 inhibitor therapy in animal models of type 1 diabetes [202], cardiac ischemia/reperfusion-injury [203], chronic myocardial infarction [204], abdominal aortic aneurysm [205], Parkinson’s diseases [206], and sepsis [207,208]. Furthermore, limited data are available on the reduction of oxidative stress by DPP-4 inhibition in humans. Shah *et al.* found reduced 3-nitrotyrosine levels in isolated human pancreatic cells after treatment with linagliptin [209], which agrees with the findings in gliptin-treated type 2 diabetic patients [210]. In humans it remains unclear whether DPP-4 inhibitor-dependent reduction of oxidative stress is independent of glucose-lowering effects. However, there is clear evidence for glucose-independent reduction of oxidative stress by DPP-4 inhibition in different animal models.

DPP4 inhibition has been shown to reduce oxidative stress in various disease models. Reports on diabetes [211,212], atherosclerosis [192,193], sepsis [207,208], and neurological disease [213] can be found in the literature. AMP-activated protein kinase (AMPK) is an important regulator of oxidative stress in the vasculature, more specifically in endothelial cells [214]. Activation of AMPK via GLP-1 receptor signaling has been shown to reduce oxidative stress in cardiomyocytes and reduces activation of NADPH oxidase [215]. On the other hand, suppression of protein kinase C (PKC)/NFκB-dependent Nox activation/upregulation might also be responsible [211,212]. For GLP-1 independent action of DPP-4 inhibition on reduction of oxidative stress, it has been proposed that DPP-4 is an adenosine deaminase (ADA)-binding protein and regulates the subcellular localization and activity of this enzyme, which has known immunomodulatory functions [216,217]. ADA activity also leads to increased inosine levels with subsequent hypoxanthine formation and thereby provides the substrate for the pro-oxidative enzyme xanthine oxidase (XO) [208]. Furthermore, several other protein targets were described for DPP-4 such as caveolin-1, kidney Na^+^/H^+^ ion exchanger 3, thromboxane A_2_ receptor, CXCR4, CXCL12 (SDF-1), fibronectin, and many more [179], most of them being involved in the regulation of inflammation. Immunomodulation by DPP-4 seems to be critical for antioxidant properties of DPP-4 inhibitors.

Since various cell types and tissues are affected by DPP-4 inhibition and also by GLP-1, it is difficult to determine which signaling pathway is predominantly responsible for reduction of oxidative stress in a specific disease model. Most of the studies that investigated the effects of DPP-4 inhibition on oxidative burst performed no experiments with genetic or pharmacological inhibition of the GLP-1 receptor. This limitation prevents a differentiation between DPP-4- and GLP-1-dependent effects. Future studies on cell-specific GLP-1 receptor knock-out animals are needed to draw a clearer picture of the complex interaction of DPP-4 and GLP-1. The following sections will focus on the antioxidant effects of DPP-4 inhibition and GLP-1 analog supplementation in atherosclerosis and sepsis.

#### 3.3.1. Gliptins and GLP-1 in Atherosclerosis

As described above, cardiovascular diseases are closely linked to oxidative stress and inflammation. Endothelial function is a reliable predictor of future cardiovascular events and is directly linked to the burden of oxidative stress in the vessel wall as well as in the blood (e.g., activation state of circulating immune cells) [12]. Recent studies demonstrate that walking distance and critical limb ischemia correlate with the activation state and production of ROS of circulating leukocytes in patients with peripheral artery disease [218,219,220]. Impaired endothelial function leads to inadequate vasodilation and increased disposition for infiltration of inflammatory cells. There is convincing evidence that atherosclerosis can be regarded an inflammatory disease [221].

Matsubara *et al.* investigated the effects of the DPP-4 inhibitor sitagliptin in an animal model of atherosclerosis [192]. They used ApoE^−/−^ mice on a high-fat diet. Sitagliptin treatment reduced atherosclerotic lesions, improved endothelial function, and reduced infiltration of CD68^+^ cells into the vascular wall. Vascular inflammation was significantly reduced by sitagliptin treatment, which was proven by reduced mRNA levels of several pro-inflammatory cytokines (IL-6, IL-1β, and TNF-α). Similar reduction of inflammation was found in cultured human monocytes. The authors also showed the anti-inflammatory effects of a GLP-1 analog (envisaged by reduced IL-6), which was additive to the beneficial effects of sitagliptin. This *in vitro* experiment demonstrated that the anti-inflammatory effects of DPP-4 inhibitor and GLP-1 analog were not connected to each other. Shah *et al.* made similar observations in LDLr^−/−^ and ApoE^−/−^ mice by using alogliptin treatment [193]. They demonstrated nicely the reduction of chemotaxis and monocyte activation by DPP-4 inhibitor therapy in both models of atherosclerosis. A major limitation of the study is that it does not differentiate between GLP-1- and DPP-4-dependent effects [193]. Besides the beneficial effects of DPP-4 inhibition on atherosclerosis, anti-atherosclerotic effects of GLP-1 supplementation were also demonstrated in animal models. GLP-1 therapy reduced vascular inflammation and increased plaque stability [222]. Others demonstrated improved endothelial function in ApoE^−/−^ mice [223]. Since oxidative stress, derived from inflammatory monocytes, is a major trigger for endothelial dysfunction [23], the beneficial effects of GLP-1 in this context might rely on inhibition of this cell type. This hypothesis is supported by a recent publication reporting on reduced oxidative stress in human monocytes after exendin-4 incubation [224]. Furthermore, the antioxidant capacity (superoxide dismutase activity) in these cells was increased by exendin-4, which could be blocked by the PKA inhibitor H89 [224]. Others attributed the anti-inflammatory effects of GLP-1 to the modulation of NFκB-activity via PKA-dependent signaling pathways [225]. GLP-1 supplementation and DPP-4 inhibition induce protective effects on vascular function in animal models of atherosclerosis. Both reduce vascular inflammation, a main trigger for oxidative stress in the vasculature. Since the GLP-1 receptor and DPP-4 are expressed in endothelial cells, it may be suggested that both can improve endothelial function by direct effects. Indeed, improved function of the endothelial NO synthase in response to GLP-1 analog treatment was demonstrated, followed by reduced activation of endothelial cells via the PI3 kinase/Akt-signaling pathway [226]. Likewise, an activation of the cAMP/PKA-signaling pathway but also the cGMP-signaling pathway was reported for GLP-1 analogs [227,228]. Also, for the DPP-4 inhibitor alogliptin, potent vasodilatory effects were described based on a Src-Akt-eNOS-dependent nitric oxide release [229]. Similar vasodilatory effects were described for linagliptin in an eNOS and soluble guanylyl cyclase-dependent fashion [208]. Furthermore, studies with HUVECs revealed the inhibitory effects of GLP-1 analog treatment on mRNA expression of Nox subunits gp91 an p22^phox^ [230].

Future studies and the use of cell-specific knock-out animals (DPP-4 and GLP-1 receptor) are needed to differentiate between the DPP-4- and GLP-1-dependent effects on vascular oxidative stress and inflammation in models of atherosclerosis.

#### 3.3.2. Gliptins and GLP-1 in Sepsis and Chronic Inflammatory Disease

Sepsis is an inflammatory disease that affects the whole organism. Depending on the immune status of a patient, simple pneumonia can expand to a “systemic inflammatory response syndrome” (SIRS), which is a severe, life-threatening condition. An average mortality of 40% makes it a leading cause of death in the European Union [231]. Because of antibiotic resistance, the use of invasive procedures, and an aging population, sepsis has become more frequent and the absence of efficient causal therapies confers high importance and priority to future research on the septic pathomechanisms but also on more promising therapeutic interventions [232].

Endotoxins (e.g., lipopolysaccharide, LPS) are responsible for the pathogenesis of sepsis in humans and animals. They are part of the outer membrane of gram-negative bacteria and trigger the pathophysiological effects (e.g., circulatory disorders). Hypotension, impaired oxygen utilization, lactic acidosis, and aggravated blood flow in the microcirculation are characteristic of sepsis, by which multiple organ failure is caused [233,234,235]. Previous studies (animal and human) could show a correlation between sepsis and endothelial dysfunction, in which oxidative stress and endothelium-derived mediators (e.g., NO, prostacyclin) are involved [236,237,238]. The oxidative burst is a key feature of neutrophils and monocytes/macrophages, which play a pivotal role in host defense. NADPH oxidase isoform 2 is generating superoxide anion radicals in response to stimuli like LPS (gram-negative bacteria) or zymosan-A (fungi). Chronic granulomatous disease (CGD) underlines the importance of Nox2-derived superoxide anion radicals for host defense. In these patients Nox2 is dysfunctional because of a genetic mutation of the gp91^phox^ gene and they are highly susceptible to infections [239]. Studies on critically ill patients revealed the protective effects of antioxidant therapy with ascorbate and α-tocopherol [240]. It reduced the risk of organ dysfunction and duration of hospitalization in these patients [240], whereas studies on other antioxidants revealed no beneficial effects [241]. The reason for these conflicting results might be that ROS formation is needed for host defense against bacteria.

A global reduction of oxidative stress in sepsis seems not to be the perfect solution to improve survival. The most promising strategy would reduce the overshooting inflammatory response in septic patients but leave the basal host defense intact. Therefore, the question is whether systemic anti-inflammatory therapy of sepsis could counteract the overshooting immune response and might improve survival. The CORTICUS trial investigated whether hydrocortisone application, as a general suppressor of inflammation, might improve survival in septic patients. In this trial no significant improvement of survival in patients suffering from septic shock was found [242]. Furthermore, statins have anti-inflammatory properties and a recent trial tested the use of rosuvastatin in patients suffering from acute respiratory distress syndrome (ARDS). The results were disappointing and statin therapy failed to improve clinical outcome [243].

**Figure 2:**
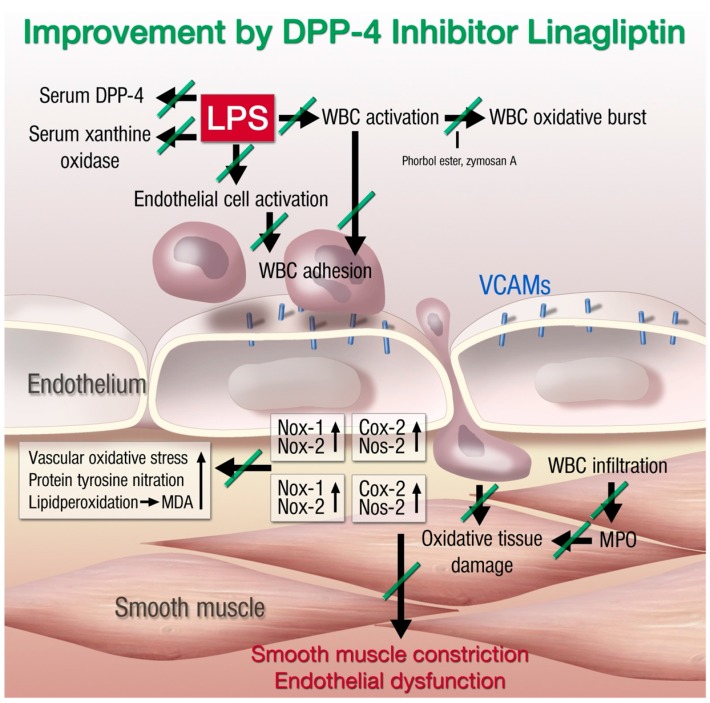
Proposed mechanisms of lipopolysaccharide (LPS)-induced vascular dysfunction and improvement by linagliptin therapy. LPS treatment activates white blood cells (WBC, envisaged by increased oxidative burst), increases serum levels of xanthine oxidase (XO), increases DPP-4 serum activity, and activates vascular cells (detected by expression of endothelial adhesion molecules and inducible cyclooxygenase (Cox-20). This leads to the infiltration of WBC to the vascular wall (detected by aortic FACS analysis for myelomonocytic cells, inducible nitric oxide synthase (Nos-2), Nox2, and myeloperoxidase (MPO) expression) and oxidative damage of the vasculature (Nox1 expression, ROS formation, 3-nitrotyrosine levels, and lipid peroxidation by malondialdehyde (MDA)). Finally, the tissue damage results in smooth muscle constriction and endothelial dysfunction. The green lines on the arrows define the inhibitory effects of linagliptin on septic complications. Adapted from [208]. With permission by Oxford University Press. Copyright © 2012, Oxford University Press.

The immunomodulatory actions of DPP-4 inhibitors and GLP-1 analogs have already been discussed in general and for models of atherosclerosis. Our group investigated the anti-inflammatory and antioxidant effects of DPP-4 inhibition and GLP-1 supplementation in models of endotoxemia. For induction of endotoxemia, the LPS injection model (10 mg/kg) was used, whereas a higher dose of LPS (17 mg/kg) was used to induce partial mortality. In a first study, we were able to show that endothelial dysfunction was severely impaired in endotoxemic rats [208]. This finding was associated with increased oxidative stress in whole blood, vessel walls, and hearts of the animals. Oral treatment with the DPP-4 inhibitor linagliptin strongly improved endothelial function and accordingly reduced oxidative stress levels. Interestingly, the reduced oxidative stress in the vascular wall was accompanied by less infiltration of CD11b^+^ cells and reduced myeloperoxidase protein expression. Our results revealed two different mechanisms for reduced oxidative stress under endotoxemic conditions in the vascular wall: (1) linagliptin treatment prevents expression of leukocyte adhesion molecules like vascular adhesion molecule-1 (VCAM-1) and thereby reduces endothelial cell activation; and (2) linagliptin directly reduces LPS-induced activation of polymorphonuclear neutrophils (PMN), which was reflected by attenuated adhesion to endothelial cells and oxidative burst. In summary, DPP-4 inhibition reduces the inflammatory state of circulating leukocytes as well as the pro-inflammatory phenotype of the vascular wall and improves the function of endothelial cells, all of which ameliorates vascular function under endotoxemic conditions (Figure 2).

**Figure 3:**
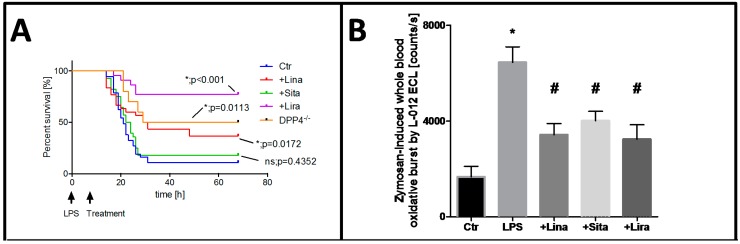

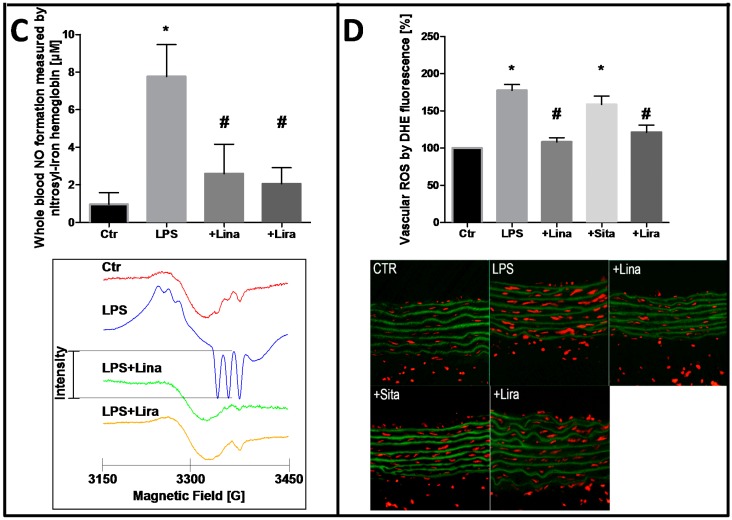
Protective effects of dipeptidyl peptidase-4 inhibition and glucagon-like peptide-1 analog supplementation in an animal model of LPS-induced endotoxemia. (**A**) Survival of animals was recorded and interpreted by Kaplan–Meier curves; (**B**) Whole blood oxidative burst upon stimulation with the fungal endotoxin zymosan A was measured by chemiluminescence using the luminol analog L-012; (**C**) iNOS-derived nitric oxide was determined in whole blood by measurement of nitrosyl-iron hemoglobin by electron paramagnetic resonance spectroscopy; (**D**) Vascular ROS formation was measured in aortic cryo-sections by dehydrothidium (DHE)-dependent oxidative fluorescence microtopography. Data are mean ± SEM of experiments with 19–36 mice (**A**) or at least three rats per group (**B**–**D**). * *p* < 0.05 *vs.* Ctr; **^#^** *p* < 0.05 *vs.* LPS. Adapted from [207]. With permission by Springer-Verlag Berlin Heidelberg. Copyright © 2015, Springer.

These promising results encouraged us to investigate the impact of DPP-4 inhibition and GLP-1 supplementation on the survival of endotoxemic mice [207]. Pre- as well as post-treatment with linagliptin and the GLP-1 analog liraglutide improved survival of endotoxemic animals significantly (Figure 3A). As a proof of concept, we tested the survival of LPS-treated DPP-4^−/−^ mice and found that these mice are also protected from endotoxic shock-dependent death [207]. Ku *et al.* found similar results in DPP-4^−/−^ rats and postulated that increased GLP-1 levels are responsible for the improved survival [244]. In line with these murine data, the oxidative burst in whole blood of LPS-treated rats was significantly increased and normalized by DPP-4 inhibition and GLP-1 supplementation (Figure 3B). In accordance with this observation, the nitrosyl-iron hemoglobin (Hb-NO) signal was measured by electron spin resonance (EPR) spectroscopy in whole blood as a direct read-out for increased iNOS activity in LPS-treated animals, which was increased in endotoxemic rats and normalized by linagliptin and liraglutide therapy (Figure 3C). As a marker of vascular oxidative stress, the dihydoethidium fluorescence signal was increased in the vascular wall of LPS-treated rats and normalized by linagliptin and liraglutide treatment (Figure 3D). In summary, these data nicely show the anti-inflammatory and antioxidant potential of DPP-4 inhibitors and GLP-1 analogs, but also reveal substantial differences between the different DPP-4 inhibiting drugs that might be related to their binding affinity and specific location in the active site of DPP-4 [207,208].

A known adverse side effect of DPP-4 inhibition in humans is an increased risk for infections like nasopharygitis (risk ratio, 1.2 (95% CI, 1.0–1.4)) or urinary tract infection (risk ratio 1.5 (95% CI, 1.0–2.2)). These results from a meta-analysis reflect the abovementioned immunomodulatory effects of DPP-4 inhibition [245]. Furthermore, the GLP-1 analog exendin-4 reduced inflammation in a non-alcoholic steatohepatitis (NASH) animal model by decreasing the infiltration of macrophages (CD68^+^, F4/80^+^) [246]. Sitagliptin improved inflammation and fibrosis in methionine/choline-deficient diet-induced steatohepatitis [247]. NASH is a liver inflammatory disease, sharing several features with atherosclerosis [248]. Besides atherosclerosis, sepsis, and NASH, DPP-4 inhibitors and GLP-1 analogs exert immunomodulatory effects in various models of chronic inflammatory diseases such as colitis [249], asthma [250], chronic obstructive lung disease (COPD) [251], and rheumatoid arthritis [252].

## 4. Immunomodulation as a Therapeutic Strategy

As already outlined above, inflammation represents an independent risk factor for the development of cardiovascular disease. C-reactive protein (CRP) is an acute phase protein and indicates inflammatory processes. According to the PROVE IT-TIMI 22 trial of patients with acute coronary syndrome after initiation of statin therapy, the risk of recurrent myocardial infarction or coronary death was significantly elevated in patients with a high hsCPR (>2 mg/L) compared to patients with low hsCPR levels [253]. The cytokine IL-17 was demonstrated to induce death of human endothelial cells, contributing to plaque destabilization and acute coronary syndrome by disruption of the blood-brain-barrier and activation of NADPH oxidase in brain endothelial cells [254,255]. These adverse effects were suppressed by administration of an IL-17A blocking antibody or by antioxidant therapy [255]. IL-17 induces endothelial cell activation, expression of endothelial adhesion molecules, followed by adhesion and infiltration of neutrophils [256], providing the rational for beneficial effects of therapy with a soluble TNF-α receptor antibody (etanercept) on angiotensin-II induced vascular superoxide production and hypertension [257,258]. The immunosuppressive drug methotrexate (MTX) is used in treatment of cancer and autoimmune disease. Rheumatoid arthritis patients suffer from chronic inflammation and have an increased risk for cardiovascular events. A meta-analysis revealed the antirheumatic drug to be protective against cardiovascular disease in patients with chronic inflammation [259]. Studies on myocardial infarction in dogs showed reduced infarction size by methotrexate treatment [260]. Based on these interesting results, the ongoing TETHYS trial, which investigates the effects of MTX therapy on myocardial infarction with ST-segment elevation, was initiated [261]. An additional clinical trial, which faces the effect of immunosuppression by MTX on the cardiovascular system, is the ongoing CIRT trial (Cardiovascular Inflammation Reduction trial). Patients with myocardial infarction and either type 2 diabetes mellitus or metabolic syndrome will be treated with low-dose MTX or a placebo [262]. Another target currently under investigation for immunomodulation in cardiovascular disease is IL-1β. Animal studies revealed that blockade of IL-1β improves endothelial regrowth, reduces neointima formation, and thereby prevents restenosis following carotid denudation [263]. Furthermore, it ameliorates cardiac remodeling and reduces cardiomyocyte apoptosis after experimental acute myocardial infarction [264]. The ongoing CANTOS trial examines the cardiovascular outcome after blockade of IL-1 β by canakinumab in post myocardial infarction patients [265]. There is convincing evidence for a contribution of inflammation to cardiovascular disease and immunomodulation is a promising new therapeutic approach to treat cardiovascular disease. Nevertheless, it is challenging to find a specific target and tool for modulating the inflammatory cascade. The ongoing clinical trials will provide more answers for these questions.

## 5. Conclusions

Endothelial dysfunction is an early hallmark of most cardiovascular disease in general [266] and coronary heart disease in particular [13], as well as related future cardiovascular events. Oxidative stress is associated with cardiovascular disease [1,2] but also represents a prognostic marker of future cardiovascular events [12]. Therefore, oxidative stress must be considered a trigger of cardiovascular events; it probably contributes to the progression of cardiovascular disease and represents an attractive target for its therapy [267]. According to more recent data, there is a close correlation between oxidative stress and inflammation in the vasculature [8,28,218,268], making both of them independent triggers/risk factors for the progression of cardiovascular disease and future cardiovascular events [12,36]. Since large clinical trials on chronic oral, systemic, unspecific antioxidant therapy failed to display beneficial effects on cardiovascular events [269], the use of source and cell (organelle)-specific compounds or activators of intrinsic antioxidant systems represents a more promising strategy [122]. Another attractive attempt might be the exploitation of the antioxidant and anti-inflammatory properties of established cardiovascular drugs [5]. Screening for candidates with potent anti-inflammatory effects could represent important additional criteria for the development of cardiovascular drugs in the future. Comparison of drugs with similar primary effects (e.g., blood pressure lowering) but with or without pleiotropic anti-inflammatory and antioxidant effects will allow us to study the importance of these pleiotropic effects. Also, prescreening of patients for markers of inflammation and/or oxidative stress will help us to develop or find the most efficient drug or drug combination for the treatment of the patients in an individual way (in the sense of personalized medicine).

The results obtained with GLP-1 supplementation and DPP-4 inhibition in models of endotoxemia are quite promising, but they remind us of the enthusiasm about statins as a new treatment strategy in sepsis and their failure in large clinical trials [270]. Animal trials and small clinical trials showed convincing evidence for a mortality reduction by statin therapy, which also relied on immunomodulatory effects [271]. Unfortunately, these results could not be reproduced in a large multi-center trial [243] and meta-analysis revealed no improvement of survival [272]. More research is needed to better characterize the antioxidant and anti-inflammatory effects of DPP-4 inhibition and GLP-1 supplementation in the pathogenesis of sepsis. Studies in different models of sepsis (*i.e.*, acute respiratory response syndrome or cecal ligation and puncture) and small clinical trials could shed light on this promising field of sepsis research in the future.
